# IFN-*γ* Reduces Epidermal Barrier Function by Affecting Fatty Acid Composition of Ceramide in a Mouse Atopic Dermatitis Model

**DOI:** 10.1155/2019/3030268

**Published:** 2019-01-29

**Authors:** Hiroyuki Kanoh, Asako Ishitsuka, Etsuko Fujine, Shuhei Matsuhaba, Mitsuhiro Nakamura, Hiroyasu Ito, Naoki Inagaki, Yoshiko Banno, Mariko Seishima

**Affiliations:** ^1^Department of Dermatology, Gifu University Graduate School of Medicine, 1-1 Yanagido, Gifu 501-1194, Japan; ^2^Laboratory of Pharmacology, Gifu Pharmaceutical University, 1-25-4 Daigaku-Nishi, Gifu 501-1196, Japan; ^3^Laboratory of Drug Informatics, Gifu Pharmaceutical University, 1-25-4 Daigaku-Nishi, Gifu 501-1196, Japan; ^4^Department of Informative Clinical Medicine, Gifu University Graduate School of Medicine, 1-1 Yanagido, Gifu 501-1194, Japan

## Abstract

IFN-*γ* is detected in chronic lesions of atopic dermatitis (AD); however, its specific role remains to be elucidated. An impaired stratum corneum barrier function is a hallmark of AD, and it is associated with a reduction in ceramides with long-chain fatty acids (FAs) in the stratum corneum. FA elongases, ELOVL1 and ELOVL4, are essential for the synthesis of these ceramides, together with ceramide synthase 3 (CerS3). We have previously shown that IFN-*γ*, but not other cytokines, induced the downregulation of these enzymes in cultured keratinocytes. Our aim was to investigate the *in vivo* role of IFN-*γ* in the lesional skin of AD by analyzing mouse dermatitis models. The local mRNA expression of IFN-*γ* increased in mite fecal antigen-induced AD-like dermatitis in NC/Nga mice but not in imiquimod-induced psoriasis-like dermatitis in BALB/c mice. The mRNA expression of ELOVL1 and ELOVL4 significantly decreased in AD-like dermatitis, whereas ELOVL1 increased in psoriasis-like dermatitis. The expression of CerS3 increased slightly in AD-like dermatitis, but it increased by 4.6-fold in psoriasis-like dermatitis. Consistently, the relative amount of ceramides with long-chain FAs decreased in AD-like dermatitis but not in psoriasis-like dermatitis. These results suggest that IFN-*γ* in the lesional skin may reduce ceramides with long-chain FAs by decreasing the expression of ELOVL. Thus, IFN-*γ* may contribute to the chronicity of AD by impairing barrier function.

## 1. Introduction

IFN-*γ* is a representative Th1 cytokine, and it is involved not only in systemic but also in cutaneous immune responses such as contact hypersensitivity reactions [1–3]. Thus, IFN-*γ* has various effects on the keratinocytes to upregulate immunological functions, for example, the expression of CD54 [4], production of proinflammatory cytokines such as IL-1*α* and TNF-*α* [5], and production of Th1- and Th2-associated chemokines [3, 6]. IFN-*γ* also modulates the functions of the Langerhans cells, including the expression of the major histocompatibility complex [7] and production of cytokines and chemokines [8]. Atopic dermatitis (AD) is a chronic, pruritic, and inflammatory dermatosis with a defective epidermal permeability barrier. In chronic lesions in patients with AD, IFN-*γ* is abundantly expressed; however, the role of IFN-*γ* in the lesional skin has not been sufficiently elucidated [9–11].

The stratum corneum (SC), the outermost layer of the skin, comprises layers of corneocytes embedded in an extracellular lipid matrix and provides an essential air–liquid barrier function to the skin. Ceramide (CER) is synthesized in epidermal keratinocytes and released into the extracellular matrix of the SC, and it plays an important role in the barrier function as a major component of the extracellular lipid matrix, which provides a barrier to water loss and prevents the penetration of various compounds into the skin [12, 13]. A reduction in CER levels in the SC has been considered as one of the causes of barrier dysfunction in AD [14]. CERs consist of sphingoid moieties (sphingosine, dihydrosphingosine, phytosphingosine, or 6-hydroxy-sphingosine) and fatty acid (FA) moieties (nonhydroxy, *α*-hydroxy, or ester-linked *ω*-hydroxy) with carbon chains of various lengths, and they are categorized into 11 classes of CERs with more than 300 species. The CER in the skin is unique because of a longer FA carbon chain than that in other tissues [15]. Interestingly, the FA carbon chain length of CERs correlates with the barrier function [16, 17], and in the SC of patients with AD, levels of CERs with long-chain FAs are reduced, which correlates with barrier dysfunction [18–20]. Thus, the composition of the FAs of CERs rather than the amount of CERs is now considered important for barrier function.

CER synthase (CerS) and FA elongase, ELOVL, are critical enzymes in the synthesis of CERs with long-chain FAs. CerS catalyzes amide bond formation between a fatty acyl-CoA and a sphingoid base to produce dihydro-CER, which is then converted to CER [21]. FAs with a carbon chain length of up to 16 (≤C16 FAs) are either synthesized de novo or absorbed from food. However, >C16 FAs, which are required for the synthesis of CERs with long-chain FAs, are produced by ELOVL [22]. We have recently shown that IFN-*γ*, but not other cytokines, decreases the expression of CerS and ELOVL in cultured human keratinocytes. Furthermore, IFN-*γ* decreases CERs with long-chain FAs in three-dimensional cornified epidermal sheets. Therefore, IFN-*γ* may have adverse effects on the lipid barrier function through the reduction in the expression of CerS and ELOVL [23]. However, IFN-*γ* is a representative Th1 cytokine that counteracts Th2 immune response; thus, it is uncertain whether this proposed function of IFN-*γ* could actually be applicable to AD, a representative Th2 disease. The purpose of the present study was to investigate the *in vivo* role of IFN-*γ* in the lesional skin of AD. We examined the relationships between the expression of IFN-*γ*, expression of ELOVL and CerS, and FA composition of CER in the lesional skin by employing two types of experimental mouse dermatitis models, namely, the NC/Nga mouse model of AD induced by house dust mite fecal antigen (FAg) and the BALB/c mouse model of psoriasis induced by imiquimod (IMQ).

## 2. Materials and Methods

### 2.1. Animals

NC/Nga mice (8-week-old females) and BALB/c mice (8-week-old females) were purchased from Japan SLC Inc. (Hamamatsu, Japan) and maintained under conventional housing conditions. All animal experiments were approved by the Animal Research Committee of Gifu University Graduate School of Medicine and conducted in accordance with the guidelines of the Japanese Association for Laboratory Animal Science (1987).

### 2.2. House Dust Mite FAg Preparation

House dust mite FAg (10 mg protein/mL containing 0.5% Tween 20) was prepared from the feces of *Dermatophagoides farinae* as described previously [24].

### 2.3. FAg-Induced Dermatitis

Experiments on FAg-induced dermatitis were performed as described by Shah et al. [24] with modifications. Briefly, an FAg or vehicle (0.5% Tween 20) solution was applied to both sides of the auricles of NC/Nga mice (25 *μ*L/auricle) twice a week for 2 weeks, for a total of five times from day 0 to day 14. For the measurement of transepidermal water loss (TEWL), the FAg solution was applied to the shaved back skin (50 *μ*L/mouse). Ear thickness was measured using a dial thickness gauge (Ozaki Mfg. Co. Ltd., Tokyo, Japan). Blood was collected for IgE measurement, and the mice were sacrificed 6 h after the fifth application, because prominent IFN-*γ* expression was observed at 6 h in preliminary experiments (data not shown). The auricles were excised, quickly frozen with liquid N_2_, and stored at −80°C before use.

### 2.4. IMQ-Induced Dermatitis

Experiments on IMQ-induced dermatitis were performed as described by van der Fits et al. [25] with modifications. Briefly, commercially available IMQ cream (5%) (Beselna® cream; Mochida Pharmaceutical Co. Ltd., Tokyo, Japan) was applied daily to both sides of the auricles of BALB/c mice (31.25 mg/auricle, a daily topical dose of 62.5 mg) for 8 days, from day 0 to day 7. Because a vehicle was not available, the control mice could not be treated with any topical cream. The mice were sacrificed 24 h after the eighth application, and the auricles were excised, quickly frozen with liquid N_2_, and stored at −80°C before use.

### 2.5. TEWL Measurement

TEWL was measured using Multi Display Devices MDD 4 attached to a Tewameter® TM 300 (Courage+Khazaka, Cologne, Germany).

### 2.6. ELISA for IgE

Serum IgE concentrations were measured using the eBioscience™ Mouse IgE ELISA Ready-SET-Go!™ Kit (Affymetrix, CA, USA).

### 2.7. Cytokine and Enzyme Expression

To evaluate cytokine and enzyme expression, total RNA was extracted from the ear samples using TRIzol® Reagent (Thermo Fisher Scientific, MA, USA), and cDNA was synthesized using the PrimeScript™ RT Reagent Kit (Perfect Real Time) (Takara Bio, Kusatsu, Japan). Real-time PCR was performed using the Thermal Cycler Dice® Real Time System (Takara Bio, Kusatsu, Japan) with SYBR® Premix Ex Taq™ II (Tli RNaseH Plus) (Takara Bio, Kusatsu, Japan). The primer sequences for evaluating cytokine and enzyme gene expression are listed in Supplemental Table 1. The primer to detect IL-12 and IL-23 was designed to amplify a p40 common subunit, thus “IL-12/IL-23” means IL-12 and/or IL-23 in this study.

### 2.8. CER Nomenclature

The nomenclature for CERs was used as described previously [23]. Briefly, CER classes were expressed as a combination of FA residues and sphingoid bases, for example, CER consisting of nonhydroxy FA and sphingosine was expressed as CER[NS].

### 2.9. Lipid Extraction from the Ear and Analysis Using Liquid Chromatography-Mass Spectrometry

The ear sample was incubated overnight at 4°C with 2.5 mg/mL DISPASE® (Wako Pure Chemical Ind. Ltd., Osaka, Japan), and the epidermis was subsequently separated enzymatically from the ear. Lipids were extracted from the epidermis using a modified Bligh and Dyer procedure as described previously [26]. C17-CER (N-heptadecanoyl-D-*erythro*-sphingosine) (Avanti Polar Lipids, Alabaster, AL, USA), which does not exist in the epidermis, was added before lipid extraction as an internal standard for quantification. The lipid extract was analyzed using ultraperformance liquid chromatography (UPLC) on an ACQUITY UPLC® BEH C18 column (1.7 *μ*m, 2.1 × 100 mm) coupled to a Waters Xevo™ QTof MS mass spectrometer (Waters, Milford, MA, USA). Various CER(NS), including C14-CER[NS] (CER[NS] with C14 FA, N-myristoyl-D-erythro-sphingosine), C16 (N-palmitoyl)-CER[NS], C18 (N-stearoyl)-CER[NS], C20 (N-arachidoyl)-CER[NS], C22 (N-behenoyl)-CER[NS], C24 (N-lignoceroyl)-CER[NS], C24 : 1 (N-nervonoyl)-CER[NS], and C26 (N-hexacosanoyl)-CER[NS] (Avanti Polar Lipids, Alabaster, AL, USA), were used as standards. Chromatographic separation was performed in a positive mode consisting of phase A (water : 7 mM HCOONH_4_ = 100 : 2) and phase B (MeOH : 5 mM HCOONH_4_ = 500 : 1).

### 2.10. Statistical Analysis

All data were expressed as mean ± SD. A pair-wise comparison was performed by Student's *t*-test using statistical software ystat 2008 (Igaku Tosho Shuppan, Tokyo, Japan). A two-sided *p* value of less than 0.05 was regarded as a statistically significant difference and indicated by ^∗^. A *p* value of less than 0.01 was indicated by ^∗∗^.

## 3. Results

### 3.1. Cytokine Expression in FAg-Induced and IMQ-Induced Dermatitis

**Figure 1:**
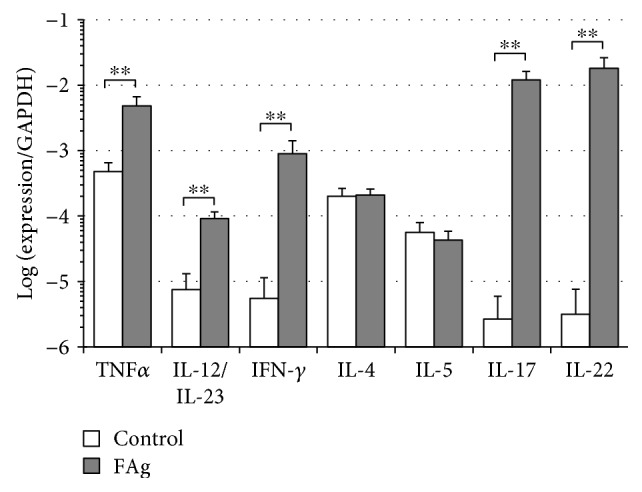
Cytokine expression in FAg-induced dermatitis: upregulation of IFN-*γ*. FAg or vehicle solution was applied to the auricles of NC/Nga mice twice a week for five times. Six hours after the fifth application on day 14, total RNA was extracted from the whole auricles and the expression of cytokines was determined using quantitative RT-PCR. “IL-12/IL-23” means IL-12 and/or IL-23 because p40, a common subunit to IL-12 and IL-23, was amplified. Expression of each cytokine is presented as a value relative to GAPDH mRNA. Data are presented as mean ± SD in nine (FAg) or ten (vehicle) mice and are from one experiment representative of two independent experiments. Two-sided Student's *t-*test was used for pair-wise comparisons. ^∗^ *P* < 0.05 and ^∗∗^ *P* < 0.01.

The NC/Nga mouse has been widely used as an AD model as it spontaneously develops AD-like skin inflammation [27]. We and another group have previously reported that repeated application of house dust mite extract or FAg onto the skin of NC/Nga mice also causes AD-like dermatitis and leads to an increase in mRNA expression of IFN-*γ* as well as Th2 cytokines such as IL-4, IL-5, and IL-13, effectively and reproducibly [24, 28]; thus, we explored FAg-induced dermatitis in the NC/Nga mouse ear. An eczematous skin lesion was observed by the fourth application of FAg at the latest, and on day 14 at the fifth application, ear thickness, transepidermal water loss (TEWL), and serum total IgE level significantly increased (Supplemental Figure 1). Six hours after the last application of FAg, mRNA expression of IFN-*γ* increased by 163-fold in the AD-like dermatitis as compared with that in control mouse skin, and the expression of TNF-*α*, IL-12/IL-23, IL-17, and IL-22 also increased (Figure 1 and Supplemental Table 2a). Since IFN-*γ* production is stimulated by IL-12, and IL-17 and IL-22 production is stimulated by IL-23 [29], the increase in “IL-12/IL-23” should reflect an increase in both IL-12 and IL-23. Although the expression of IL-4 and IL-5 did not increase (Figure 1 and Supplemental Table 2a), when the expression was evaluated 12 h after the last application in preliminary experiments, IL-4 and IL-5 also increased (data not shown) as reported previously [24, 28].

**Figure 2:**
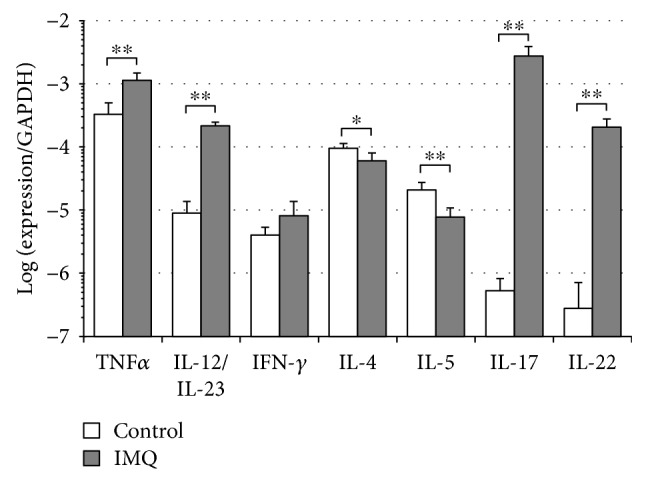
Cytokine expression in IMQ-induced dermatitis: no change in IFN-*γ*. IMQ cream was applied daily to the auricles of BALB/c mice for 8 times. As control, nothing was applied. Twenty-four hours after the eighth application on day 7, total RNA was extracted from the whole auricles and the expression of cytokines was determined by quantitative RT-PCR. “IL-12/IL-23” means IL-12 and/or IL-23 because p40, a common subunit to IL-12 and IL-23, was amplified. Expression of each cytokine is presented as a value relative to GAPDH mRNA. Data are presented as mean ± SD in six mice per group and are from one experiment representative of two independent experiments. Two-sided Student's *t-*test was used for pair-wise comparisons. ^∗^ *P* < 0.05 and ^∗∗^ *P* < 0.01.

Daily application of IMQ onto the BALB/c mouse skin causes psoriasis-like skin inflammation [25]. Although IFN-*γ* is detected in the lesional skin in humans with psoriasis [30–32], mRNA expression of IFN-*γ* in mice with IMQ-induced dermatitis is only modestly elevated, and the protein levels of IFN-*γ* are not altered [33, 34]. Therefore, we employed this model for the assessment of the role of IFN-*γ* in comparison with FAg-induced dermatitis. We confirmed that dermatitis was observed after the fifth application of IMQ onto the BALB/c mouse ear. Twenty-four hours after the eighth application of IMQ on day 7, the mRNA expression of TNF-*α*, IL-12/IL-23, IL-17, and IL-22 increased in psoriasis-like dermatitis as compared with that in control mouse skin. However, no significant increase in the expression of IFN-*γ* or of IL-4 and IL-5 was observed (Figure 2 and Supplemental Table 2b). As is the case in FAg-induced dermatitis, the increase in “IL-12/IL-23” should reflect an increase in both IL-12 and IL-23 in IMQ-induced dermatitis.

### 3.2. Changes in mRNA Expression of ELOVL and CerS in FAg-Induced and IMQ-Induced Dermatitis

Seven ELOVL isozymes and six CerS isozymes exist, and each isozyme exhibits a characteristic substrate specificity toward FA carbon chain length [21, 22, 35]. Among them, ELOVL1 and ELOVL4 play a critical role in the production of CERs with very long-chain FAs; ELOVL1 elongates C18–C26 FAs, which are further elongated to >C26 FAs by ELOVL4 [36]. A deficiency in ELOVL4 causes lethal dysfunction of the epidermal barrier [37–39]. CerS3 prefers ≥C26 FAs as a substrate. In CerS3 knockout mice, severe TEWL and early postnatal lethality are observed [40]. Thus, we examined the expression of ELOVL and CerS isozymes in the dermatitis models.

**Figure 3:**
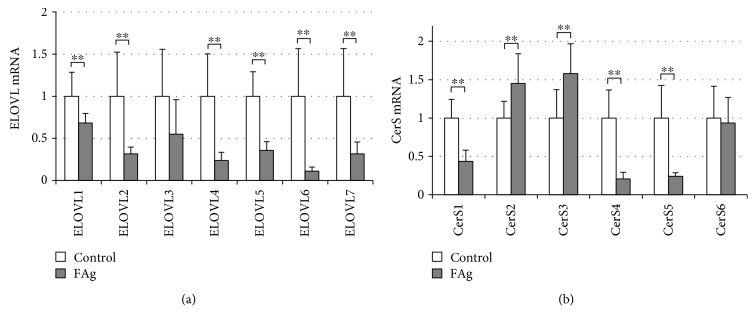
Reduced expression of ELOVL and CerS in FAg-induced dermatitis. Dermatitis was induced by FAg as described in Figure 1, and the expression of ELOVL and CerS isozymes was determined by quantitative RT-PCR. (a) Changes in the expression of ELOVL isozymes by repeated application of FAg. (b) Changes in the expression of CerS isozymes by repeated application of FAg. Data are values relative to control. All data are presented as mean ± SD in nine (FAg) or ten (vehicle) mice and are from one experiment representative of two independent experiments. Two-sided Student's *t-*test was used for pair-wise comparisons. ^∗^ *P* < 0.05 and ^∗∗^ *P* < 0.01.

In the control NC/Nga mouse ear in experiments with house dust mite FAg, the mRNA of ELOVL4 was prominently expressed, followed by that of ELOVL6, whereas the expression of the other isozymes was very low (Supplemental Table 3a). However, it should be noted that the relative mRNA expression may be affected by the primer design for each enzyme. The expression of ELOVL4 substantially decreased by 76.2 ± 9.6% in FAg-induced dermatitis compared with that in control mouse skin, and the expression of the other ELOVL isozymes except for ELOVL3 also significantly decreased, with the highest inhibition in ELOVL6 (Figure 3(a) and Supplemental Table 4a). Among the six isozymes, the mRNA of CerS4 was prominently amplified in the control NC/Nga mouse ear by the primer sets used here (Supplemental Table 3b). In FAg-induced dermatitis, the expression of CerS1, CerS4, and CerS5 decreased, whereas the expression of CerS2 and CerS3 mildly increased with statistical significance (Figure 3(b) and Supplemental Table 4b).

**Figure 4:**
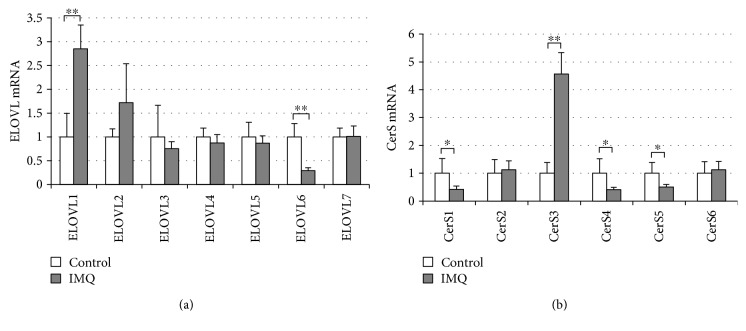
Minimal changes in the expression of ELOVL and CerS in IMQ-induced dermatitis. Dermatitis was induced by IMQ as described in Figure 2, and the expression of ELOVL and CerS isozymes was determined by quantitative RT-PCR. (a) Changes in the expression of ELOVL isozymes by repeated application of IMQ. (b) Changes in the expression of CerS isozymes by repeated application of IMQ. Data are values relative to control. All data are presented as mean ± SD in six mice per group and are from one experiment representative of two independent experiments. Two-sided Student's *t-*test was used for pair-wise comparisons. ^∗^ *P* < 0.05 and ^∗∗^ *P* < 0.01.

In the control BALB/c mouse ear in experiments with IMQ, the mRNA expression profile of the ELOVL isozymes was similar to that observed in the control NC/Nga mouse ear; mRNA of ELOVL4 was prominently amplified, followed by ELOVL6, whereas the expression of the other isozymes was very low (Supplemental Table 5a). In contrast to FAg-induced dermatitis, the expression level of ELOVL4 remained unchanged in IMQ-induced dermatitis. Among the isozymes, only the expression of ELOVL6, which elongates C16 FA to C18 FA [36], decreased, and that of ELOVL1 notably increased (Figure 4(a) and Supplemental Table 6a). As is the case with the ELOVL isozymes, the mRNA expression profile of the CerS isozymes was similar to that observed in the control NC/Nga mice; mRNA of CerS4 was prominently amplified (Supplemental Table 5b). In IMQ-induced dermatitis, the expression of CerS1, CerS4, and CerS5 had a tendency to decrease, whereas the expression of CerS3 increased by 4.6-fold (Figure 4(b) and Supplemental Table 6b).

### 3.3. Differences in FA Carbon Chain Length of CER in FAg-Induced and in IMQ-Induced Dermatitis

**Figure 5:**
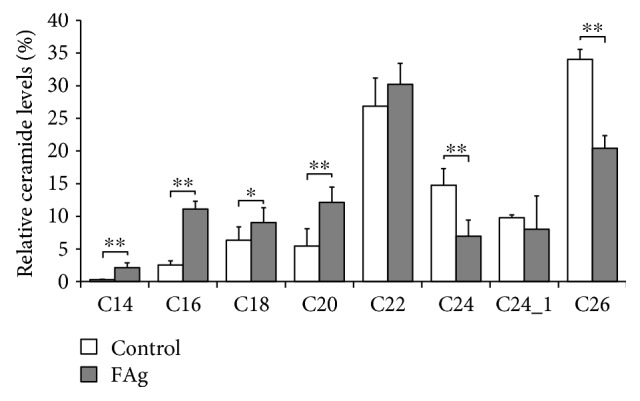
Reduction in CER[NS] with long-chain FAs in FAg-induced dermatitis. FAg or vehicle solution was applied to the auricles of NC/Nga mice twice a week for 5 times. Six hours after the fifth application on day 14, auricles were excised, and the epidermis was enzymatically separated. Lipids were extracted from the epidermis and analyzed using liquid chromatography–mass spectrometry. Data are presented as mean ± SD of the percentage of the total amount of C14-CER to C26-CER in nine (FAg) or five (vehicle) mice and are from one experiment representative of two independent experiments. Two-sided Student's *t-*test was used for pair-wise comparisons. ^∗^ *P* < 0.05 and ^∗∗^ *P* < 0.01.

**Figure 6:**
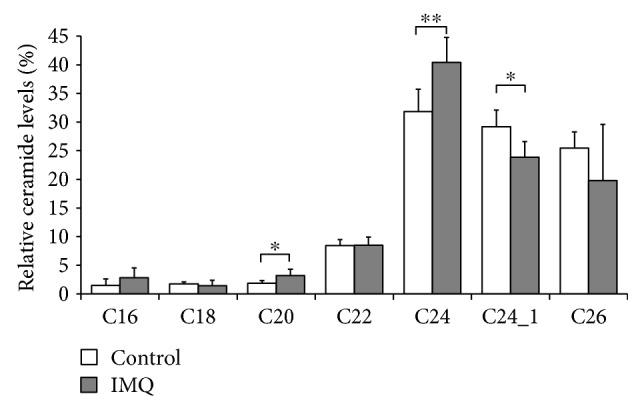
Minimal change in the FA carbon chain length of CER[NS] in IMQ-induced dermatitis. IMQ was applied to the auricles of BALB/c mice daily for 8 times. Twenty-four hours after the eighth application on day 7, the auricles were excised, and the epidermis was enzymatically separated. Lipids were extracted from the epidermis and analyzed using liquid chromatography–mass spectrometry. Data are presented as mean ± SD of the percentage of the total amount of C16-CER to C26-CER in six (IMQ) or five (control) mice and are from one experiment representative of two independent experiments. Two-sided Student's *t-*test was used for pair-wise comparisons. ^∗^ *P* < 0.05 and ^∗∗^ *P* < 0.01.

To examine whether the FA carbon chain length of CERs is affected by a decreased expression of ELOVL and CerS, lipids were extracted from the epidermis of the ear, and the relative amount of CERs consisting of nonhydroxy FA and sphingosine (CER[NS]) was analyzed using liquid chromatography–mass spectrometry. CER[NS] is the major CER class in the SC; furthermore, the FA carbon chain length of CER[NS] is related to barrier function [16–18]. The relative amount of CER[NS] with a long-chain FA, that is, C24-CER[NS] and C26-CER[NA], decreased in FAg-induced dermatitis, whereas the amount of C14-CER[NS], C16-CER[NS], and C20-CER[NS] increased compared with that in control mice (Figure 5 and Supplemental Table 7a). In contrast, only minor changes in the FA carbon chain length of CER[NS] were observed in IMQ-induced dermatitis (Figure 6 and Supplemental Table 7b), which were consistent with the minor changes in the expression of ELOVL and CerS (Figure 4).

## 4. Discussion

We have previously shown that IFN-*γ*, but not other cytokines, reduces the expression of ELOVL and CerS in cultured keratinocytes [23]. Furthermore, IFN-*γ* decreases CERs with long-chain FAs in three-dimensional cornified epidermal sheets [23], suggesting that IFN-*γ* reduces the FA carbon chain length of CERs in the SC through the reduction of the ELOVL and CerS expression levels in keratinocytes. In the present study, FAg-induced AD-like lesions and IMQ-induced psoriasis-like lesions showed contrasting cytokine profiles: the expression of IFN-*γ* was significantly elevated in FAg-induced dermatitis but not in IMQ-induced dermatitis. However, the two models resembled each other with respect to other cytokine profiles, making the *in vivo* assessment of IFN-*γ* function reasonable. Our assessment of enzyme expressions and the FA carbon chain length of CERs in this cytokine environment, in combination with our previous findings in cultured keratinocytes [23], strongly suggests that IFN-*γ* may be involved in the decrease of CERs with long-chain FAs in the epidermis through the reduction in ELOVL expression in FAg-induced AD-like dermatitis.

The regulation of ELOVL and CerS in the skin is closely related to keratinocyte differentiation. The mRNA expression of ELOVL4 and CerS3 increases during keratinocyte differentiation [41]. Vitamin D receptor activation induces keratinocyte differentiation, and accordingly, vitamin D receptor silencing reduces the mRNA expression of ELOVL3 and ELOVL4 [42]. On the other hand, in a mouse model of AD induced by repeated application of hapten, the downregulation of ELOVL1 and ELOVL4 and the resultant decrease in CERs with long-chain FAs are also observed [43]. Recently, it was shown that the expression of ELOVL1 is reduced in the lesional skin of human AD by immunohistochemical staining [44]. However, the regulation mechanisms of these enzymes in an inflamed skin are poorly understood. In the present study, the expression of ELOVL1 and ELOVL4 was also downregulated in FAg-induced AD-like dermatitis, and the relative amount of CERs with long-chain FAs, C24-CER[NS] and C26-CER[NS], consistently decreased. We have shown that these phenomena may be elicited by IFN-*γ*. Although the expression levels of CerS2 and CerS3, which prefer long-chain FAs as a substrate [21], were mildly elevated in FAg-induced dermatitis, those of CERs with long-chain FAs were decreased. This is probably because the decrease in long-chain FA production due to the reduction in ELOVL1 and ELOVL4 levels may have outweighed the slight increase in the CerS2 and CerS3 expression levels. The increase in CerS2 and CerS3 also suggests that the expression of CerS isozymes is not solely regulated by IFN-*γ in vivo*. The complexity in the regulation mechanism of these enzymes is also presumed by the fact that the degree of reduction in ELOVL and CerS isozymes by IFN-*γ* depends on the differentiation status of cultured keratinocytes [23].

Changes in the expression of ELOVL and CerS isozymes in IMQ-induced dermatitis, in which the level of IFN-*γ* did not significantly change, also indicates that the expression of these enzymes is not regulated solely by IFN-*γ in vivo*. In particular, the mRNA expression of ELOVL1 and CerS3 increased significantly in IMQ-induced dermatitis. Although the mechanism underlying the upregulation remains to be clarified, some factors other than cytokine may be involved. In addition, the level of CERs with long-chain FAs remained unchanged in spite of the increased expression of ELOVL1 and CerS3. Since the expression level of ELOVL1 is very low compared with that of ELOVL4 (Figure 4(a)), the production of very long-chain FAs may predominantly depend on ELOVL4. Alternatively, the decreased expression of ELOVL6, which elongates C16 FA to C18 [35], might have reduced the substrate FAs for CerS3.

Although we have not examined it in the present study, free FAs and cholesterol are also important components of the SC extracellular lipid matrix [12]. In a mouse model of AD, not only ≥C26-CERs but also ≥C26 free FAs decrease in the analysis of whole epidermal lipids [45]. Examination of SC lipids in the lesional skin of AD patients reveals that the carbon chain length of free FAs are reduced as well as that of CERs [20, 44, 46]. The reduced mRNA expression of ELOVL1 and ELOVL4 in FAg-induced AD-like dermatitis in the present study and the reduced protein expression of ELOVL1 in the SC of human AD patients shown by Danso et al. [44] suggest that the carbon chain length of free FAs in the SC may also be regulated by a common synthetic pathway, and free FAs may also play important roles in the skin barrier function.

The role of IFN-*γ* in the pathophysiology of AD is controversial. AD was initially considered in the context of a Th1/Th2 immunity balance with Th2 skewing and suppression of the Th1 axis [47]. The level of IFN-*γ*-producing T cells is decreased in peripheral blood samples from patients with AD [48–50], and systemic recombinant IFN-*γ* therapy is effective to some extent [51, 52]. In cultured human epidermal sheets or reconstructed epidermal equivalents, the amount of CER is increased by IFN-*γ* [53, 54] and this upregulation is inhibited by IL-4 [53]. Overall, these facts suggest that IFN-*γ* is involved in antagonizing the pathogenesis of AD. Nonetheless, local skin analysis shows an abundant expression of IFN-*γ* [9–11]. Until recently, AD was characterized as a biphasic T cell-mediated disease; a Th2 signal predominates in the acute phase, and a Th2 to Th1 switch promotes disease chronicity in the skin. Recently, the involvement of Th17 and Th22 cytokines in addition to Th1 and Th2 cytokines has been documented, and two endotypes of AD have been proposed: type 2 immune response and nontype 2 immune response including Th17-, Th22-, and Th1-driven inflammation [55–57]. In particular, the chronic phase is characterized by an intensification of immune activation rather than an immune switch; that is, the progression to chronic disease is associated with an upregulation of Th22 and Th2 immune axes as well as significant increases in Th1-related products in the lesional skin [11]. Although JAK transduces the signals of IFN-*γ* as well as Th2 cytokines [58, 59], excellent results in patients with AD responding to treatment with the topical JAK inhibitor have been reported [60, 61]. In this context, the presence of IFN-*γ* in the lesional skin may not necessarily be a counteracting force to the Th2 immune response but may be involved in the pathogenesis of AD.

We acknowledge that this study has limitations. First, mite FAg-induced dermatitis in a NC/Nga mouse does not necessarily reflect a chronic lesion of AD. However, the NC/Nga mouse has been widely used as an AD model because it spontaneously develops AD-like skin inflammation [27]; thus, FAg-induced dermatitis, in which IFN-*γ* as well as Th2 cytokines were highly expressed, may mimic some aspects of the chronic lesion of AD. Second, since we analyzed the FA carbon chain length of CER in the whole epidermis but not in the SC, the relative amount of CERs with various carbon chain lengths does not completely reflect the composition of the SC CERs. In addition, C16-CER is the dominant molecular species in the basal layer of the epidermis [62]. However, suprabasal keratinocytes undergoing differentiation increase the expression of ELOVL4 and CerS3 and thus the production of >C16-CERs [21, 41], most of which would become a component of SC CERs. Therefore, the analysis of whole epidermal lipids would be an acceptable strategy for the analysis of SC CERs in a mouse model of AD [45]. And third, an actual evaluation of the direct effect of IFN-*γ* on ELOVL in keratinocytes was not performed because the present study analyzed the relationships among cytokine expression, enzyme expression, and the FA composition of CERs in animal models. However, on the basis of our present findings in combination with our previous findings that IFN-*γ* reduces the expression of these enzymes via its receptor on cultured keratinocytes [23], we strongly suggest that the observed phenomena in this study indicates that IFN-*γ* acts on keratinocyte to reduce ELOVL expression.

## 5. Conclusions

In the present study employing animal models, we showed that IFN-*γ*, a representative Th1 cytokine, may decrease CERs with long-chain FAs in the lesional skin in AD through the reduction in the expression of ELOVL in the epidermis. This may be one of the mechanisms of the impaired barrier function in the chronic lesion of AD, in which various types of T helper cells including Th1 cells are simultaneously activated. Thus, IFN-*γ* may contribute to the chronicity of AD by impairing barrier function.

## Data Availability

The data used to support the findings of this study are available from the corresponding author upon request.

## Abbreviations

AD: Atopic dermatitis
CER: Ceramide
CER[NS]: Ceramide composed of nonhydroxy fatty acid and sphingosine
C16-CER: CER with C16 (palmitoyl) fatty acid
CerS: Ceramide synthase
FA: Fatty acid
FAg: Fecal antigen
IMQ: Imiquimod
SC: Stratum corneum
TEWL: Transepidermal water loss.

## Supplementary Materials

Supplementary 1 Figure S1: ear thickness, TEWL, and serum IgE level in FAg-induced dermatitis. FAg or vehicle solution was applied to the shaved back skin (for TEWL) and auricles of NC/Nga mice twice a week for 5 times. On day 14, ear thickness, TEWL, and serum IgE level were measured. Data are presented as mean ± SD in nine (FAg) or five (vehicle) mice, and are from one experiment representative of two independent experiments. Two-sided Student's *t*-test was used for pair-wise comparisons. ^∗^ *P* < 0.05 and ^∗∗^ *P* < 0.01.

Supplementary 2 Table S1: sequences of primers used in quantitative RT-PCR. Table S2a: cytokine expression in FAg-induced dermatitis: upregulation of IFN-*γ*. Table S2b: cytokine expression in IMQ-induced dermatitis: no change in IFN-*γ*. Table S3a: relative mRNA expression of ELOVL isozymes in control NC/Nga mice. Table S3b: relative mRNA expression of CerS isozymes in control NC/Nga mice. Table S4a: reduced expression of ELOVL in FAg-induced dermatitis. Table S4b: reduced expression of CerS in FAg-induced dermatitis. Table S5a: relative mRNA expression of ELOVL isozymes in control BALB/c mice. Table S5b: relative mRNA expression of CerS isozymes in control BALB/c mice. Table S6a: expression of ELOVL isozymes in IMQ-induced dermatitis. Table S6b: expression of CerS isozymes in IMQ-induced dermatitis. Table S7a: reduction in CER[NS] with long-chain FAs in FAg-induced dermatitis. Table S7b: minimal change in the FA carbon chain length of CER[NS] in IMQ-induced dermatitis.
