# Supplementary material for: IFN-γ Reduces Epidermal Barrier Function by Affecting Fatty Acid Composition of Ceramide in a Mouse Atopic Dermatitis Model
Source: J Immunol Res. 2019 Jan 29;2019:3030268. doi: 10.1155/2019/3030268 (PMC6374817; doi:10.1155/2019/3030268)
Supplement: Supplementary 2 — Table S1: sequences of primers used in quantitative RT-PCR. Table S2a: cytokine expression in FAg-induced dermatitis: upregulation of IFN-γ. Table S2b: cytokine expression in IMQ-induced dermatitis: no change in IFN-γ. Table S3a: relative mRNA expression of ELOVL isozymes in control NC/Nga mice. Table S3b: relative mRNA expression of CerS isozymes in control NC/Nga mice. Table S4a: reduced expression of ELOVL in FAg-induced dermatitis. Table S4b: reduced expression of CerS in FAg-induced dermatitis. Table S5a: relative mRNA expression of ELOVL isozymes in control BALB/c mice. Table S5b: relative mRNA expression of CerS isozymes in control BALB/c mice. Table S6a: expression of ELOVL isozymes in IMQ-induced dermatitis. Table S6b: expression of CerS isozymes in IMQ-induced dermatitis. Table S7a: reduction in CER[NS] with long-chain FAs in FAg-induced dermatitis. Table S7b: minimal change in the FA carbon chain length of CER[NS] in IMQ-induced dermatitis. [file 3030268.f2.docx]

**Supplemental Table 1: Sequences of primers used in quantitative RT-PCR.**

gene sequence

ELOVL1　 Forward: 5’-CCAGGGCAGGAGTCTCAAAG-3’

Reverse: 5’-AACAGCCTCCATCCTGGCTAA-3’

ELOVL2　 Forward: 5’-AGCTCACAGTACCAGCCAGCTTTAG-3’

　　　　 Reverse: 5’-TTCGTAGCTCTGCATGGTGTGTC-3’

ELOVL3　 Forward: 5’-TGGTCCTTCTTCCTGGCAAT-3’

Reverse: 5’-AGGATGATGAAGGCCGTGT-3’

ELOVL4　 Forward: 5’-TTGTGCGTTGAGATTCCATTCC-3’

Reverse: 5’-AGCCAGTACAGAATGTTCGCAAAG-3’

ELOVL5　 Forward: 5’-CAGGGCCCAGAGCTTGTTAGTTTA-3’

　　　　 Reverse: 5’-ACCAAGGGTGCCATCAGATTTC-3’

ELOVL6　 Forward: 5’-CAGTTAAGTGTGGGTCTGAGCATGA-3’

　　　　 Reverse: 5’-GTGATTTCGCTGTGAGGCTTGA-3’

ELOVL7　 Forward: 5’-GGCTTGGACTGACAGCATGG-3’

Reverse: 5’-GAGGACTGCACATGTGTACTATGGA-3’

CerS1　 Forward: 5’-GGTCAGATGCGTGAACTGGAA-3’

Reverse: 5’-GGATAGAGTCCTGGATGGCTGAA-3’

CerS2　 Forward: 5’-CGTGTCTATGCCAAAGCCTCA-3’

　　　　 Reverse: 5’-GTCTGGTAGAAATGTTCCAAGGTG-3’

CerS3　 Forward: 5’-ACCCTGGCTGAACTTTGTGTGA-3’

Reverse: 5’-GCCTTTGTACCTCCGGGTATCTTA-3’

CerS4　 Forward: 5’-CATGACTGCTCCGACTACCTG-3’

　　　　 Reverse: 5’-GAATATGAGGCGCGTGTAGAA-3’

CerS5　 Forward: 5’-TCTCCGTGAGGATGCTGTTTG-3’

　　　 Reverse: 5’-GTGTCATTGGGTTCCACCTTATTG-3’

CerS6 Forward: 5’-CACCTGGGCAGACCTGAAGA-3’

Reverse: 5’-TGGCACATGGTTTGGCTATGA-3’

GAPDH　 Forward: 5’-TGTGTCCGTCGTGGATCTGA-3’

　　　　 Reverse: 5’-TTGCTGTTGAAGTCGCAGGAG-3’

IFN-γ Forward: 5’-CGGCACAGTCATTGAAAGCCTA-3’

Reverse: 5’-GTTGCTGATGGCCTGATTGTC-3’

TNF-α Forward: 5’-GCCAGGAGGGAGAACAGAAACTC-3’

Reverse: 5’-GGCCAGTGAGTGAAAGGGACA-3’

IL-4　 Forward: 5’-ACGGAGATGGATGTGCCAAAC-3’

　　 Reverse: 5’-AGCACCTTGGAAGCCCTACAGA-3’

IL-5 Forward: 5’-TGAGGCTTCCTGTCCCTACTCATAA-3’

Reverse: 5’-TTGGAATAGCATTTCCACAGTACCC-3’

IL-12/IL-23* Forward: 5’-CCGGTCCAGCATGTGTCAA-3’

Reverse: 5’-CTTCACCATGTCATCTGTGGTCTTC-3’

IL-17　　 Forward: 5’-GAAGGCCCTCAGACTACCTCAA-3’

　　　　 Reverse: 5’-TCATGTGGTGGTCCAGCTTTC-3’

IL-22　 Forward: 5’-TCCAGCAGCCATACATCGTC-3’

　 Reverse: 5’-CTTCCAGGGTGAAGTTGAGCA-3’

*The primer was designed to amplify p40: a common subunit to IL-12 and IL-23.

**Supplemental Table 2a. Cytokine expression in FAg-induced dermatitis: upregulation of IFN-γ.**

Control FAg P

TNFα 4.76E-4 ± 1.78E-4 48.2E-4 ± 18.3E-4 <0.0001

IL-12/23 7.47E-6 ± 5.62E-6 91.2E-6 ± 24.2E-6 <0.0001

IFN-γ 5.49E-6 ± 5.98E-6 896E-6 ± 528E-6 0.0030

IL-4 2.01E-4 ± 0.611E-4 2.08E-4 ± 0.504E-4 0.2697

IL-5 5.63E-5 ± 2.35E-5 4.31E-5 ± 1.50E-5 0.1677

IL-17 2.67E-6 ± 3.20E-6 12100E-6 ± 4170E-6 <0.0001

IL-22 3.14E-6 ± 4.46E-6 18600E-6 ± 8740E-6 <0.0001

Data in Figure 1 are shown. Two-sided Student’s *t-*test was used for pair-wise comparisons.

**Supplemental Table 2b. Cytokine expression in IMQ-induced dermatitis: no change in IFN-γ.**

Control IMQ P

TNFα 3.29E-4 ± 1.67E-4 11.2E-4 ± 3.64E-4 0.0007

IL-12/23 8.99E-6 ± 4.73E-6 215E-6 ± 31.3E-6 <0.0001

IFN-γ 4.02E-6 ± 1.30E-6 8.12E-6 ± 5.58E-6 0.1102

IL-4 9.49E-5 ± 1.88E-5 6.00E-5 ± 2.04E-5 0.0116

IL-5 2.07E-5 ± 0.670E-5 0.770E-5 ± 0.308E-5 0.0015

IL-17 5.29E-7 ± 3.00E-7 27600E-7 ± 11200E-7 0.0001

IL-22 2.79E-7 ± 4.37E-7 2050E-7 ± 722E-7 0.0001

Data in Figure 2 are shown. Two-sided Student’s *t-*test was used for pair-wise comparisons.

**Supplemental Table 3a. Relative mRNA expression of ELOVL isozymes in control NC/Nga mice**

ELOVL1 107E-5 ± 30.2E-5

ELOVL2 2.64E-5 ± 1.38E-5

ELOVL3 855E-5 ± 476E-5

ELOVL4 140000E-5 ± 70000E-5

ELOVL5 2240E-5 ± 651E-5

ELOVL6 10400E-5 ± 5880E-5

ELOVL7 1500E-5 ± 846E-5

Data of control NC/Nga mice in Figure 3a are shown. The expression of each isozyme is presented as a value relative to GAPDH mRNA.

**Supplemental Table 3b. Relative mRNA expression of CerS isozymes in control NC/Nga mice**

CerS1 2.09E-4 ± 0.50E-4

CerS2 24.2E-4 ± 5.27E-4

CerS3 62.4E-4 ± 23.1E-4

CerS4 635E-4 ± 233E-4

CerS5 43.6E-4 ± 18.5E-4

CerS6 35.6E-4 ± 14.7E-4

Data of control NC/Nga mice in Figure 3b are shown. The expression of each isozyme is presented as a value relative to GAPDH mRNA.

**Supplemental Table 4a. Reduced expression of ELOVL in FAg-induced dermatitis**

Control FAg *p*

ELOVL1 1.00 ± 0.28 0.68 ± 0.11 0.0060

ELOVL2 1.00 ± 0.53 0.32 ± 0.08 0.0013

ELOVL3 1.00 ± 0.56 0.55 ± 0.41 0.0635

ELOVL4 1.00 ± 0.50 0.24 ± 0.10 0.0003

ELOVL5 1.00 ± 0.29 0.36 ± 0.10 <0.0001

ELOVL6 1.00 ± 0.56 0.11 ± 0.05 0.0002

ELOVL7 1.00 ± 0.57 0.32 ± 0.14 0.0026

Data in Figure 3a are shown. Data are values relative to control. Two-sided Student’s *t-*test was used for pair-wise comparisons.

**Supplemental Table 4b. Reduced expression of CerS in FAg-induced dermatitis**

Control FAg *p*

CerS1 1.00 ± 0.24 0.44 ± 0.14 <0.0001

CerS2 1.00 ± 0.22 1.45 ± 0.39 0.0055

CerS3 1.00 ± 0.37 1.58 ± 0.39 0.0014

CerS4 1.00 ± 0.37 0.21 ± 0.09 <0.0001

CerS5 1.00 ± 0.42 0.24 ± 0.05 <0.0001

CerS6 1.00 ± 0.41 0.93 ± 0.33 0.7099

Data in Figure 3b are shown. Data are values relative to control. Two-sided Student’s *t-*test was used for pair-wise comparisons.

**Supplemental Table 5a. Relative mRNA expression of ELOVL isozymes in control BALB/c mice**

ELOVL1 58.9E-5 ± 29.1E-5

ELOVL2 1.98E-5 ± 0.33E-5

ELOVL3 1410E-5 ± 936E-5

ELOVL4 56300E-5 ± 10400E-5

ELOVL5 1090E-5 ± 335E-5

ELOVL6 8060E-5 ± 2230E-5

ELOVL7 495E-5 ± 92.3E-5

Data of control BALB/c mice in Figure 4a are shown. The expression of each isozyme is presented as a value relative to GAPDH mRNA.

**Supplemental Table 5b. Relative mRNA expression of CerS isozymes in control BALB/c mice**

CerS1 2.35E-4 ± 1.25E-4

CerS2 20.7E-4 ± 10.2E-4

CerS3 38.1E-4 ± 14.7E-4

CerS4 453E-4 ± 237E-4

CerS5 56.5E-4 ± 21.7E-4

CerS6 30.1E-4 ± 12.6E-4

Data of control BALB/c mice in Figure 4b are shown. The expression of each isozyme is presented as a value relative to GAPDH mRNA.

**Supplemental Table 6a. Expression of ELOVL isozymes in IMQ-induced dermatitis**

Control IMQ P

ELOVL1 1.00 ± 0.49 2.85 ± 0.50 <0.0001

ELOVL2 1.00 ± 0.17 1.72 ± 0.82 0.0624

ELOVL3 1.00 ± 0.67 0.75 ± 0.15 0.3949

ELOVL4 1.00 ± 0.19 0.87 ± 0.18 0.2408

ELOVL5 1.00 ± 0.31 0.87 ± 0.15 0.3717

ELOVL6 1.00 ± 0.28 0.29 ± 0.06 0.0001

ELOVL7 1.00 ± 0.57 0.32 ± 0.14 0.9467

Data in Figure 4a are shown. Data are values relative to control. Two-sided Student’s *t-*test was used for pair-wise comparisons.

**Supplemental Table 6b. Expression of CerS isozymes in IMQ-induced dermatitis**

Control IMQ P

CerS1 1.00 ± 0.53 0.42 ± 0.12 0.0253

CerS2 1.00 ± 0.49 1.13 ± 0.32 0.6095

CerS3 1.00 ± 0.39 4.57 ± 0.77 <0.0001

CerS4 1.00 ± 0.52 0.41 ± 0.09 0.0210

CerS5 1.00 ± 0.38 0.51 ± 0.09 0.0120

CerS6 1.00 ± 0.42 1.13 ± 0.30 0.5571

Data in Figure 4b are shown. Data are values relative to control. Two-sided Student’s *t-*test was used for pair-wise comparisons.

**Supplemental Table 7a. Reduction in CER[NS] with long-chain FAs in FAg-induced dermatitis**

Control FAg P

C14-CER 0.28 ± 0.068 2.13 ± 0.77 0.0002

C16-CER 2.53 ± 0.67 11.1 ± 1.19 <0.0001

C18-CER 6.33 ± 2.03 9.04 ± 2.28 0.0472

C20-CER 5.44 ± 2.68 12.1 ± 2.38 0.0004

C22-CER 26.9 ± 4.33 30.2 ± 3.25 0.1257

C24-CER 14.8 ± 2.53 6.96 ± 2.49 0.0001

C24_1-CER 9.77 ± 0.47 8.03 ± 5.08 0.4695

C26-CER 34.0 ± 1.51 20.4 ± 1.92 <0.0001

Data in Figure 5 are shown. Data are presented as percentage of the total amount of C14-CER to C26-CER. Two-sided Student’s *t-*test was used for pair-wise comparisons.

**Supplemental Table 7b. Minimal change in the FA carbon chain length of CER[NS] in IMQ-induced dermatitis**

Control FAg P

C16-CER 1.48 ± 1.13 2.81 ± 1.73 0.1760

C18-CER 1.74 ± 0.35 1.41 ± 0.98 0.5003

C20-CER 1.84 ± 0.49 3.19 ± 1.11 0.0131

C22-CER 8.45 ± 1.04 8.49 ± 1.44 0.9618

C24-CER 31.8 ± 3.90 40.4 ± 4.34 0.0076

C24_1-CER 29.2 ± 2.91 23.9 ± 2.72 0.0120

C26-CER 25.5 ± 2.82 19.8 ± 9.81 0.2468

Data in Figure 6 are shown. Data are presented as percentage of the total amount of C16-CER to C26-CER. Two-sided Student’s *t-*test was used for pair-wise comparisons.
